# 

**Published:** 1891-02

**Authors:** 


### CONTENTS FOR FEBRUARY, 1891.

ORIGINAL COMMUNICATIONS.

Pathology and Treatment of Later Stages of Coxitis. By HERMAN MYNTER, M. D....... 386
A Discussion of Vaginal Hysterectomy with Observations of Eleven Cases with one Death. By CHARLES A. L. REED, M, D. ........ 396
The Prevention of Purulent Conjunctivitis in the Eyes of New-Born Infants. By JACOB M. FALK, M. D........ 406

SOCIETY PROCEEDINGS.

American Public Health Association........ 414
New York Academy of Medicine — Section on Orthopedic Surgery........ 418
Buffalo Medical and Surgical Association........ 421

CLINICAL REPORT.

Fistula of the Parotid Gland. By A. L. BENEDICT, M. D........ 423

PROGRESS IN MEDICAL SCIENCE.

Laryngology and Rhinology........ 425

CORRESPONDENCE.

Synopsis of the Mütter Lectures........ 429

NEW INSTRUMENTS.

Koch's Syringe........ 434

EDITORIAL.

The Koch Secret Announced........ 435

SOCIETY MEETINGS.

Medical Society of the State of New York........ 437

PERSONAL.

Dr. William C. Krauss—Dr. Frank W. Low........ 441

BOOK REVIEWS.

A Manual of Modern Surgery........ 441
Causes and Treatment of Sterility in Both Sexes, and Fecundation by Artificial Methods 443
The Physicians' Hand-Book for 1891........ 444
Books and Pamphlets Received........ 444

MISCELLANY.

The Mattison Prize........ 447
Civil Service Examination........ 448
Announcement........ 448

